# The Thalamus as a Low Pass Filter: Filtering at the Cellular Level does Not Equate with Filtering at the Network Level

**DOI:** 10.3389/fncir.2015.00089

**Published:** 2016-01-14

**Authors:** William M. Connelly, Michael Laing, Adam C. Errington, Vincenzo Crunelli

**Affiliations:** ^1^Division of Neuroscience, School of Biosciences, Cardiff UniversityCardiff, UK; ^2^Eccles Institute of Neuroscience, The John Curtin School of Medical Research, The Australian National UniversityCanberra, ACT, Australia; ^3^School of Medicine, Neuroscience and Mental Health Research Institute, Cardiff UniversityCardiff, UK; ^4^Department of Physiology and Biochemistry, University of MaltaMsida, Malta

**Keywords:** thalamus, sensory neuroscience, integrate-and-fire neuron, computational neuroscience, neural noise

## Abstract

In the mammalian central nervous system, most sensory information passes through primary sensory thalamic nuclei, however the consequence of this remains unclear. Various propositions exist, likening the thalamus to a gate, or a high pass filter. Here, using a simple leaky integrate and fire model based on physiological parameters, we show that the thalamus behaves akin to a low pass filter. Specifically, as individual cells in the thalamus rely on consistent drive to spike, stimuli that is rapidly and continuously changing over time such that it activates sensory cells with different receptive fields are unable to drive thalamic spiking. This means that thalamic encoding is robust to sensory noise, however it induces a lag in sensory representation. Thus, the thalamus stabilizes encoding of sensory information, at the cost of response rate.

## Introduction

The thalamus lies at a cross roads between the external world and the cerebral cortex (Steriade, 2003). All senses excluding olfaction pass through the thalamus, where they are subjected to some kind of processing, before being routed to the cortex. The action of the thalamus in this context has been likening to a gate, that is to say, only letting through information as dictated by higher areas (Wang et al., 2006; McAlonan et al., 2008; Saalmann and Kastner, 2011). It has also been proposed that the thalamus acts as a frequency-sensitive filter, generating spikes more easily when some time-dependent component of the sensory stimuli is correct (Heggelund et al., 2003).

The proposition that the thalamus acts as a high-pass filter has been most thoroughly explored in the vibrotactile thalamocortical system: the thalamic ventroposterior medial (VPm) nucleus and the barrel cortex. Here there is clear evidence that when the whisker is driven by a sinusoidal deflection, cortical, and thalamic spiking rates are higher with increasing stimulus frequency (Arabzadeh et al., 2003; Khatri et al., 2004). However, this effect cannot be completely ascribed to thalamic filtering, due to the velocity sensitive nature of the sensory organ (Hartings et al., 2003; Gerdjikov et al., 2010). Conversely, both the neurons of the lateral geniculate nucleus (LGN) and the primary visual cortex have most often been shown to produce low pass or band pass behavior (Hawken et al., 1996; Van Hooser et al., 2013). Again, however, this result is confounded by the fact that the retinal processing itself acts as either a low pass or band pass filter (Shapley and Victor, 1978).

The fact that individual neurons in the thalamus act as high-pass filters in terms of input rate vs. output rate is a necessary consequence of the neural membrane being a leaky capacitor. Specifically, a certain amount of charge must be delivered into a neuron in a given time to bring it to the threshold for firing and this can only be achieved when the neuron's inputs fire above certain rate. However, filtering in the context of firing rate must be considered separately from filtering in terms of what data the neural circuit is extracting from its input. That is to say, one can imagine a hypothetical neural system that codes for a particular property of a sensory stimuli, where increasing the rate of change of that property causes a significant increase in the firing rate of neurons in the system, but the system itself loses the ability to encode that property. For instance, consider a stimuli that can be in two states, and two populations of neurons coding for these two states: when one population is active the neural system is encoding one state, when the other population is active the system is encoding the other state. Finally, if both populations are active at the same time, the system encoding is ambiguous. When the stimuli is slowly changing between its two states, the neurons in each population might fire slowly, but so long as the two populations are not active at the same time, then the neural system should be able to encode the two stimuli. However, when the stimuli is rapidly switching between these two states, the neurons in our hypothetical system begin firing more rapidly. If the stimuli is switching rapidly enough the two cell populations will become active at the same time and the neural system lose encoding ability. Thus, when considered on a single cell basis, the network appears to behave as a high-pass filter (that is, the neurons fire faster as the stimulus property changes faster), but when considered from a population standpoint, the network is in fact behaving as a low-pass filter (the network loses the ability to encode the stimulus as the stimulus property changes faster).

Here we report, using a computational approach, based on a highly simplified, yet biologically reasonable model that the thalamus necessarily works to stabilize sensory representations in the presence of noise in a manner akin to a low pass filter.

## Methods

### *In vitro* electrophysiology

Wistar rats, of either sex, at postnatal days 20–30 were anesthetized with isoflurane and decapitated in accordance with the United Kingdom Animals (Scientific Procedures) Act of 1986 and local ethical committee approval. As described previously by Turner and Salt (1998) brains were rapidly removed and 300 μm-thick slices containing the dorsal lateral geniculate nuclei and an intact retinogeniculate pathway were cut in continuously oxygenated sucrose aCSF (Errington et al., 2010). Slices were incubated for at least 1 h before being transferred to the recording chamber where they were continuously perfused (~2 ml/min) with warmed (32–34°C) oxygenated recording aCSF containing the following (in mM) 125 NaCl, 5 KCl, 25 NaHCO_3_, 1.25 NaH_2_PO_4_, 1 MgCl_2_, 2 CaCl_2_, 25 glucose. Whole-cell patch-clamp recordings were made using pipettes (resistance, 2–4 MΩ) containing the following (in mM): 135 K-methylsulfonate, 10 HEPES, 10 Na-phosphocreatine, 4 MgCl2, 4 Na-ATP, 0.4 mM Na-GTP, pH 7.3, 300 mOsm. Current clamp was performed with a Multiclamp 700B preamplifier (Molecular Devices). Experimental data were filtered at 6 kHz, digitized at 20 kHz (Digidata 1322A; Molecular Devices), and acquired using pClamp 10 software (Molecular Devices). Electrical stimulation was evoked with a constant current stimulus isolator (DS3, Digitimer) through a bipolar tungsten electrode (Frederick Haer) in the presence of the GABA_A_ receptor antagonist gabazine (10 μm), and the GABA_B_ receptor antagonist 55845 (1 μm; Tocris).

### Computational modeling

**Figure 1:**
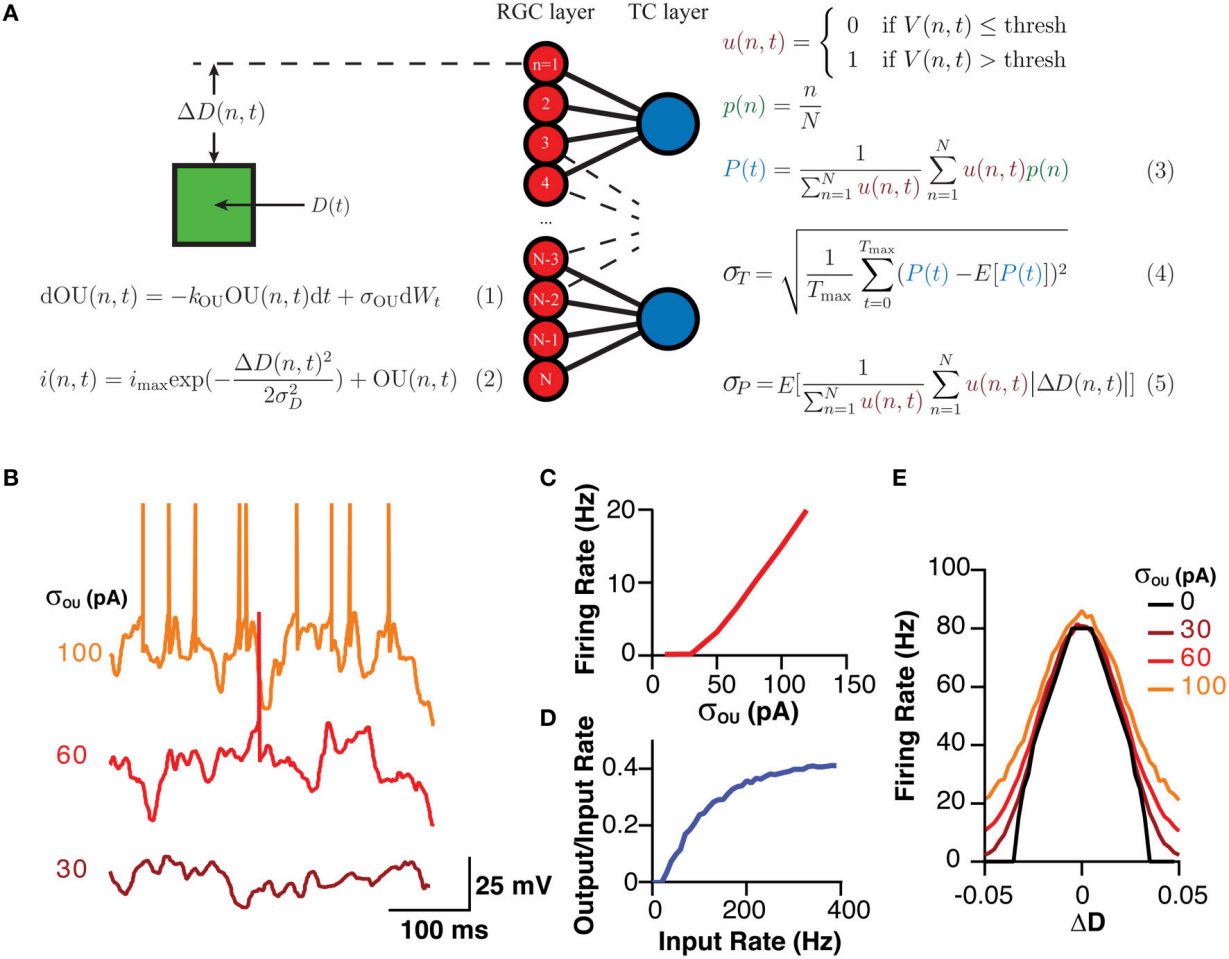
**A schematic of the model, the equations governing its behavior and its analysis and the model's typical behavior**. **(A)** A Schematic of the model and the fundamental equations dictating its behavior and analysis. Equation (1) produces the current noise while Equation (2) shows that the total current the membrane receives is the sum of the OU noise and a “sensory” current that is a Gaussian function of the difference between the cells position and stimuli. Equation (3) demonstrates how the estimator for the position of the stimuli was calculated. Equation (4) shows how σ_T_, which was used as a measure of the stability of the encoding over time, was calculated as the standard deviation of P(t). Equation (5) shows how σ_P_, a measure of the spatial accuracy of the encoding in the layer, was calculated. Note, color of equations is used to link similar terms. E[X] represented the expectation value of the expression X. **(B)** Representative examples of the membrane potential of RGCs in response to various amplitudes of OU noise. **(C)** The mean firing rate of an RGC in response to various amplitudes of OU noise. **(D)** The transfer function of TCCs, represented as the rate of retinogeniculate EPSPs against the ratio of the output (spiking rate) to the input (EPSP rate), showing the high-pass behavior of TCCs. **(E)** The sensory behavior of RGCs showing their firing rate as a function of the distance to the stimuli (ΔD) and the amplitude of the OU noise.

The network model was realized in Python 2.7. All cells were simple leaky integrate-and-fire neurons with no explicitly enforced refractory period. When Vm reached threshold, a 1 ms long spike was generated, after which Vm was reset to the resting membrane potential. The model consisted of two separate layers, a sensory layer that responded to a stimuli referred to as the retinal ganglion cell (RGC) layer, and a integrative layer, referred to as the thalamocortical cell (TCC) layer, that received input from the RGC layer (Figure 1).

For RGCs, membrane current was calculated as a sum of two currents, a noisy current and a sensory current. The noisy current was an Ornstein–Uhlenbeck process with a time constant (1/*k*_OU_) of 5 ms and an amplitude (σ_ou_) that was varied between 0 and 120 pA (Figures 1A,B,C; Destexhe et al., 2001). The sensory current was a designed to model vision, and was a current whose amplitude varied in a Gaussian fashion with distance from the visual stimuli [*D(n,t)*]. The maximum this current reached was 200 pA, which meant the maximum visually evoked firing rate for a RGC was ~80 Hz (Figures 1A,E; Croner and Kaplan, 1995). This Gaussian current had a standard deviation (σ_D_) of 2.5% of visual space. Visual space was defined as the maximum field within which RGCs could code, with 0 representing one end, and 1 representing the other. With 200 RGCs, the center of each RGC's receptive field [*p(n)*] was centered at 0.5 % of visual space from its neighbors. RGCs had a membrane resistance of 260 MΩ and a membrane capacitance of 96 pF, giving them a membrane time constant of 25 ms (O'Brien et al., 2002). RGCs had a threshold of 20 mV depolarized to rest. It should be noted that these properties intentionally give the RGCs a relatively flat frequency-response profile, allowing us to resolve the filtering properties of the TCC layer without being confounded by filtering at the retinal level.

TCCs had a membrane resistance of 70 MΩ and a membrane capacitance of 160 pF, giving them a membrane time constant of 11 ms (Crunelli et al., 1987; White and Sur, 1992). Excitatory input to TCCs was modeled as a current that synaptic input caused to instantaneously increase by 750 pA and which decayed with first-order kinetics and a time constant of 1.6 ms (Chen and Regehr, 2000). This produced a unitary excitatory postsynaptic potential (EPSP) 3.5 mV in amplitude. Spiking threshold was set at 9 mV (see Section Results).

In order to decode the information in a given layer at a given time [*P(t)*], we sought to use a maximum likelihood estimator (MLE) approach, that is to calculate the value of *D(t)* that maximizes the probability of getting a particular pattern of neural activity. However, under the assumption that the probability of any neuron in a given layer firing was an arbitrary Gaussian function of its distance to the stimuli, we were left with an expression that was mathematically uncooperative, and in our hands at least, could only be solved by numerical methods. Given this, we attempted a much simpler approach, where the position information encoded in any layer at time *t*, was simply the average *p(n)* of all spiking neurons at time *t*, given by the equation.

$$\begin{array}{l} P\left(t\right)=\;\frac{1}{\sum_{n=1}^{N}u\left(n,t\right)}\overset{N}{\underset{n=1}{\sum }}u\left(n,t\right)p\left(t\right) \end{array}$$

where *u(n,t)* is either 1 or 0 depending on whether neuron *n* is firing at time *t*, or not. Fortunately, the MLE of *P(t)* calculated numerically for all possible patterns of a small population of neurons (*n* = 24), produces values almost identical to that calculated via the mean approach (Linear regression: Slope = 0.95, *R*^2^ = 0.96, *n* = 8388608). Thus, the mean approach was used. In order to give some measure of the stability of the encoding over time we calculated the standard deviation of *P(t)* over time (σ_T_). We also sought to capture some measure of spatial accuracy of the encoding. Thus, we used the mean over time of the average distance between *p(n)* of all spiking neuron and the target (σ_P_), that is

$$\begin{array}{l} \delta_{P}=E\left[\frac{1}{\sum_{n=1}^{N}u\left(n,t\right)}\overset{N}{\underset{n=1}{\sum }}u\left(n,t\right)△D\left(n,t\right)\right] \end{array}$$

Simulations were performed with a fixed time-step of 0.5 ms. Phase (φ) information was extracted by performing a Fast Fourier Transform on *P(t)*, and the results are presented as the phase in the cell layer minus the phase of the target. Phase responses were fit with the following function:

$$\begin{array}{l} \varphi =\tan^{-1}\left(-2\pi f\tau \right) \end{array}$$

where 1/2πτ gives the corner frequency of the filter, or π/4 point. Statistical tests were performed using linear regression via Matlab R2014b (Mathworks).

## Results

**Figure 2:**
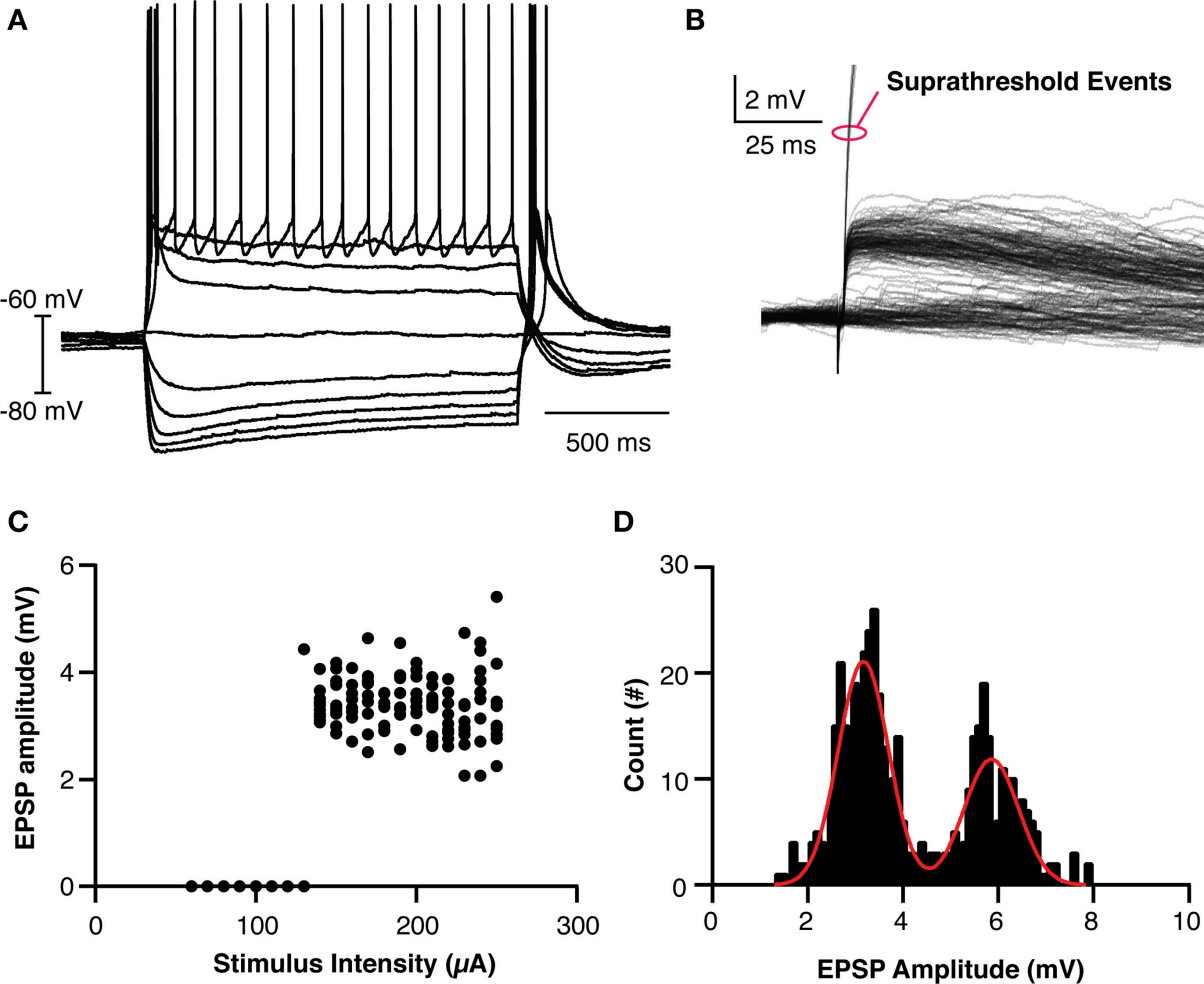
**Basic Properties of LGN thalamocortical neurons and their retinogeniculate EPSPs**. **(A)** Overlaid traces showing the response of an LGN cell to current injection. **(B)** Overlaid traces showing the recruited retinogeniculate EPSPs in response to optic nerve stimulation. Events marked with red oval are truncated spikes. **(C)** Calculated EPSP size vs. stimulation intensity for the cell shown in **(B)**, clearly showing the lack of graded recruitment of EPSP amplitude. **(D)** The histogram of EPSP amplitudes across all cells and events, clearing showing two peaks, where the amplitude of the larger peak is essentially twice the amplitude of the smaller peak. The cell in **(C)** had events only from the smaller amplitude peak.

In order to make a reasonable model of thalamic behavior, the native properties of retinogeniculate transmission was studied *in vitro*. TCCs (*n* = 26) in the dLGN were patch clamped and input from the optic tract was evoked using a ramping stimulation intensity to recruit minimal events and to investigate the threshold for action potential generation (Figures 2A,B,C). The evoked EPSPs had the hallmarks of retinogeniculate EPSPs in that they had all-or-none responses as opposed to the graded recruitment typical for corticothalamic EPSPs (Turner and Salt, 1998). Across all cells and stimulation intensities, the distribution of EPSPs amplitudes (*n* = 461 events) clearly formed two peaks, one at 3.2 mV and one at 5.9 mV (95% confidence interval 3.1–3.2 mV and 5.7–6.0 mV, respectively) and the largest subthreshold EPSP recorded was 7.9 mV (Figure 2D). We believe these peaks represent the recruitment of one and two retinogeniculate axons, respectively. As no subthreshold EPSP was ever seen with an amplitude that matched three times the single axon event, we believe this demonstrates that dLGN TCCs at rest require, on average, the coincident input of three retinal EPSPs to be driven to spike. These results fit with others who reported that one or two subthreshold unitary events could be recruited in dLGN neurons, and the development evidence that dLGN TCCs receive input from 3 to 5 RGCs (Turner and Salt, 1998; Chen and Regehr, 2000; Tavazoie and Reid, 2000; Hong and Chen, 2011).

Based on these data we developed a simple model (see Section Methods), involving 120 integrate-and-fire neurons representing a simplified RGC layer, connected to either 60, 120, or 240 TCCs, consistent with the ratio of RGC to LGN neurons (Spear et al., 1996). Cells in the RGC layer were connected in a visuotopic manner to neurons in the TCC layer, such that each TCC received input from the four (unless stated otherwise) “nearest” RGCs, while needing three coincident EPSPs to spike (Figure 1A). RGCs were driving by a sum of two currents. The first component was membrane noise modeled as an Ornstein–Uhlenbeck process, whose parameter σ_OU_ dictated the magnitude of the noise and subsequent noise induced firing rate (Figures 1B,C; Equation 1). The second current had a Gaussian receptive field with a full-width at half-maximum of ~6% of the visual field such that during complete visual activation RGCs would fire at a maximum of ~80 Hz (Figure 1E; Equation 1; Croner and Kaplan, 1995). As expected for a simple leaky integrate-and-fire neuron, when one considered the rate of EPSP input against the ratio of the output spiking rate to the input EPSP rate, each individual cell functioned as a high pass filter (Figure 1D). It is important to note, that while this model is explicitly of the visual system, it should not be assumed that we have tried to make this model replicate all the features of the visual system. Indeed, we have purposefully kept the model simple to allow it to generalize to other sensory system. Thus, while we have called the sensory layer the RGC layer, it should more be thought of as a set of sensory cells who have some arbitrary receptive field, and hence could just as well be called the “trigeminal nucleus layer” with each cell having a preferred direction of whisker deflection (Minnery et al., 2003).

**Figure 3:**
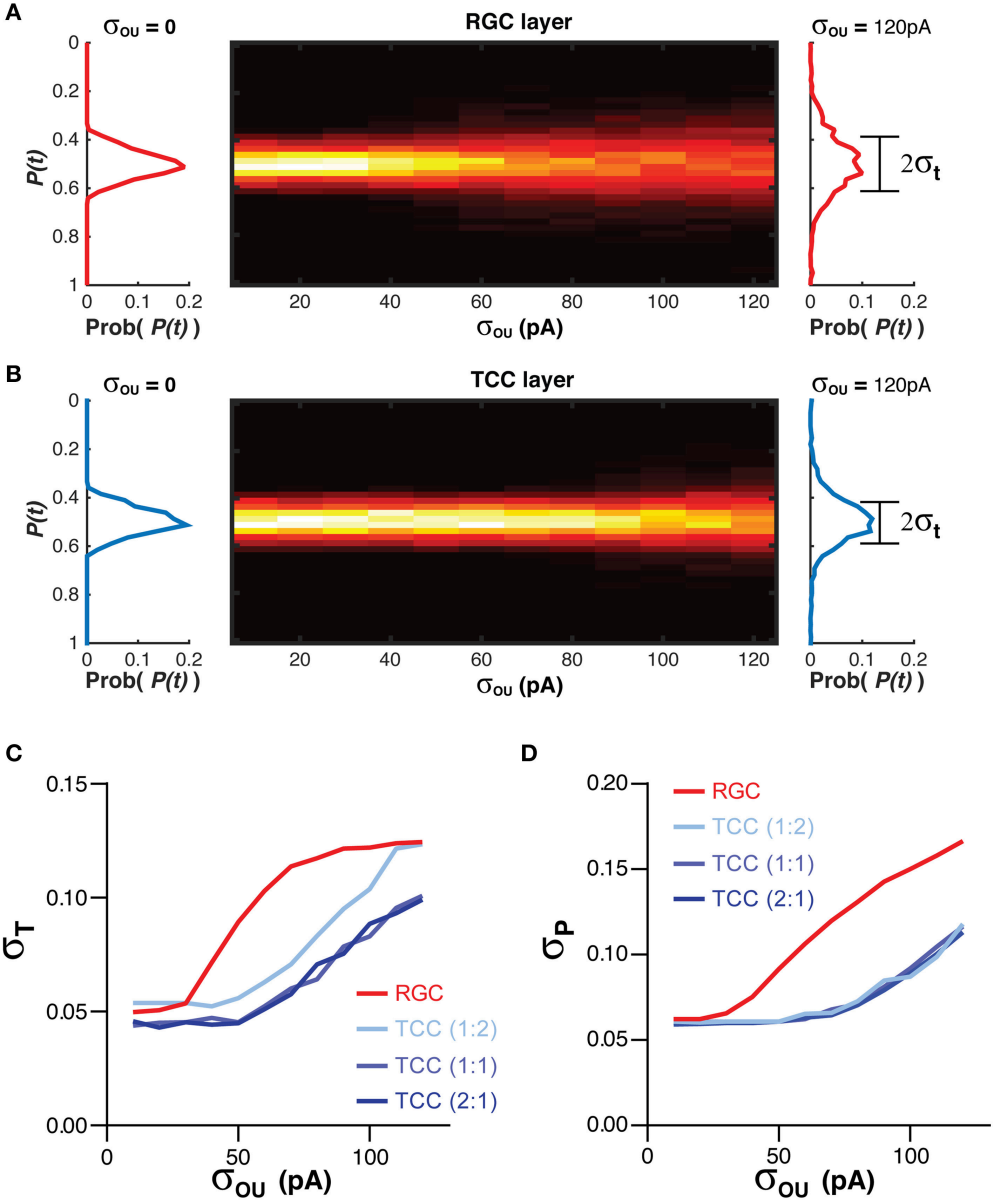
**Integration by TCCs increases the accuracy of the sensory coding in the presence of noise. (A)** To the left and right of the heat map are histograms showing the distribution of the decoded position of the stimuli [P(t)] in the RGC layer, in the presence of no retinal noise (σ_OU_ = 0) and a large degree of retinal noise (σ_OU_ = 120 pA). The central heat map shows the distribution of P(t) across a range of retinal noise. Note the broadening of the distribution of P(t) as the amplitude of the retinal noise increases. **(B)** The same data as presented in **(A)**, but for the TCC layer. Again, as the noise in the RGC layer increases, the distribution of P(t) broadens, but to lesser degree. **(C)** The effect of network architecture (the ratio of TCCs to RGCs) and retinal noise on the distribution of P(t) (σ_T_). As retinal noise increases, in both the RGC and TCC layers, σ_T_ increases, but over most of the parameter space, the TCC layer has a significantly lower value of σ_T_, that is, the encoding of position is more stable over time. **(D)** The effect of network architecture and retinal noise on the accuracy of encoding (σ_P_). Again, retinal noise causes worse performance in both layers but the thalamic layer performs better at all but the lowest levels of retinal noise. However, the ratio of TCCs to RGCs has no effect on performance.

The position information at any time for each cell layer [*P(t)*] was calculated when the simulation was exposed to a single stationary visual stimuli taking up 20% of the visual space (Figure 1; Equation 3). The standard deviation of P(t), σ_T_, acted as a measure of the stability of the representation over time (Figure 1; Equation 4). Higher values of σ_T_ are seen as an indicator of poorer network performance due to the reasonable assumption that information encoded in a network should not change if the input does not change. As the membrane noise of the RGC layer was increased, σ_T_ increased in both RGC and TCC layers (*P* < 1 × 10^−28^) (Figures 3A–C). Given that in this model TCCs receive their sole input from RGCs, it might seem reasonable to assume that the thalamic layer could only report the stimulus position as accurately as the TCC layer (receptive field half widths were almost identical between individual RGC and TCC, at 2.5 and 2%, respectively). However, apart from when RGCs had a subthreshold level of noise and the thalamic layer was made up of less cells than the RGC layer, the thalamic layer had significantly lower values of σ_T_, (*P* < 1 × 10^−12^). Furthermore, when considering σ_T_ in just TCCs, increasing the ratio of cells in the TCC layer to RGC layer (while maintaining the number of inputs that each TCC receives, meaning that each RGC projects to more TCCs) caused the values of σ_T_ to drop (*P* = 0.003), presumably due to the simple fact that there were more TCCs available to be directly driven by visually stimulate RGCs. More importantly however, the result that σ_T_ was lower in the TCC layer than the RGC layer was independent of the ratio of TCCs to RGCs over this range (Figure 3C). This clearly shows that integration by the TCC layer enhances the stability of the visual representation over time, however, how does it affect the spatial accuracy? Therefore, to measure how integration by the TCC layer affects spatial accuracy, we calculated σ_P_, a measure which was in essence the average distance between each active cell and the center of the visual target (Figure 1; Equation 5).

Increasing the amplitude of the RGC membrane noise significantly increased σ_P_ in both layers (*P* < 1 × 10^−30^), but unlike σ_T_, altering the ratio of RGCs to TCCs have no effect on σ_P_ (*P* = 0.9). Again, however, the TCC layer proved to be more accurate, having a significantly lower σ_P_ (*P* < 5 × 10^−18^; Figure 3D). Simply put, these data show that integration by the TCC layer allows the TCC layer to provide a more temporally stable and spatially accurate representation than the RGC layer, by filtering out “occasional” noise-driven spikes from the RGC layer. One could consider occasional spikes as high frequency input, not in the sense of high frequency EPSPs, but as a high frequency change in which position information that is being supplied to the TCC layer. That is, if the stimuli rapidly moved to a new position and then back to its original position, it could create spike patterns in the RGC layer very similar to noise: one or two spikes in an otherwise silent spike history. If the TCC layer does not respond to these high frequency changes in the stimuli, then we could see the TCC layer as acting like a low pass filter.

**Figure 4:**
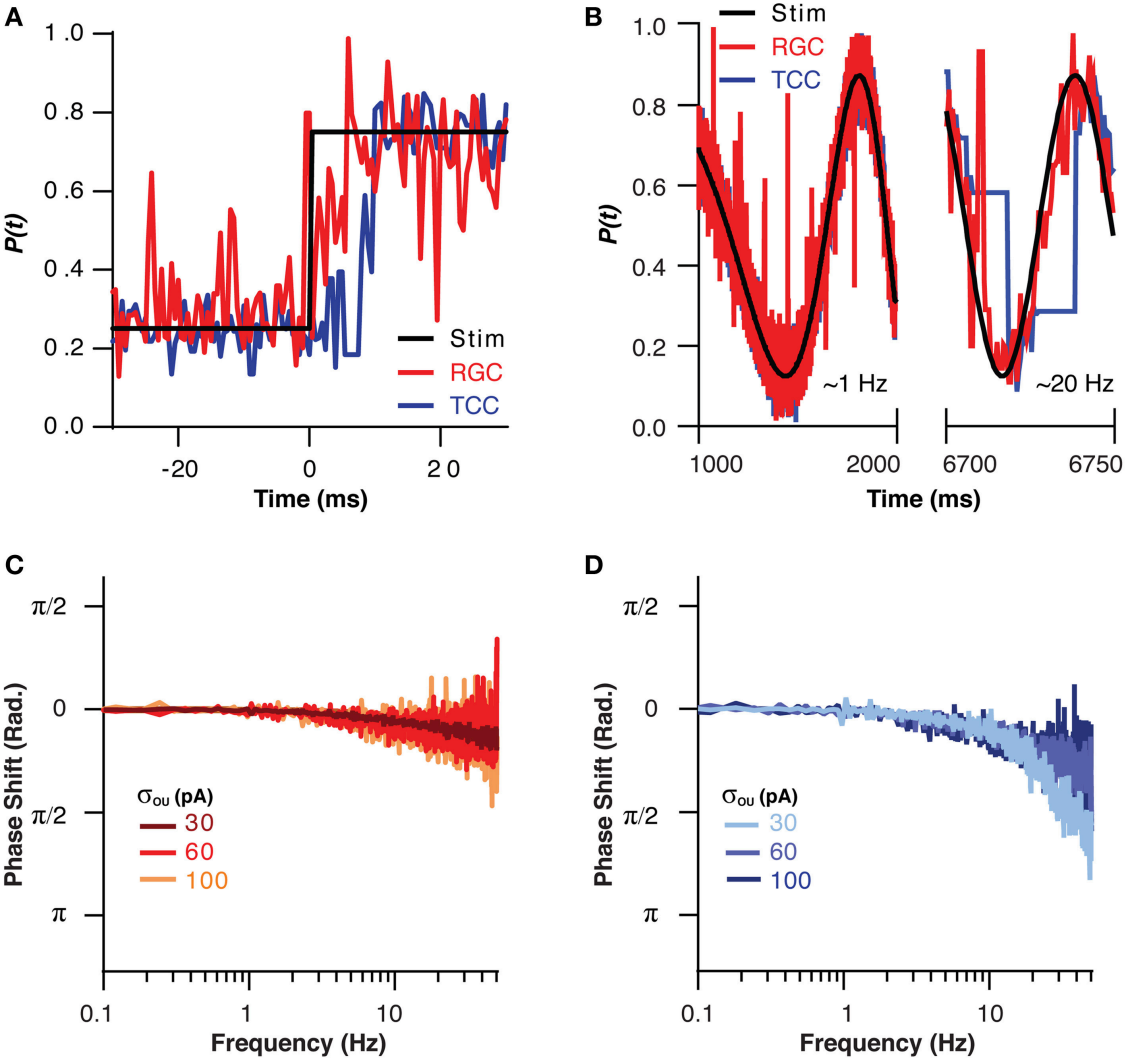
**The filtering effect of the thalamic layer comes at the cost of response rate. (A)** The position encoded by the RGC and TCC layers in response to a step change in the position of the stimuli, note the response of the TCC layer is significantly delayed relative to the response of the RGC layer. **(B)** Two sections of the response of the RGC and TCC layers to a sinusoidal input of increasing spatial frequency (σ_*ou*_ = 0.03). **(C)** The phase of the RGC layer response with respect to the stimuli, showing the minimal phase shift and general independence from the magnitude of retinal noise. **(D)** The phase of the TCC response with respect to the stimuli, showing the large phase shift than that seen in the RGC layer, and the effect that increasing retinal noise decreases the phase shift seen in the TCC layer.

If the TCC layer is in fact acting as a low pass filter for sensory information, then like all low pass filters, it must come at a cost in terms of response rate. This effect could be clearly seen when the visual stimuli was instantly stepped from one part of the visual space to another, as the RGC layer took only approximately 4 ms to encode the new position, while the TCC layer took 8 ms (Figure 4A). This effect was investigated in a more quantitative fashion by moving the visual stimuli across the visual space in a sinusoid at increasing frequencies up to 50 Hz (Figure 4B). The RGC layer was able to accurately follow the visual stimuli, and at no point did it encode beyond π/4 radians behind the stimuli (Figure 4C). The TCC layer, on the other hand, fell behind to this extent at less than 20 Hz (Figure 4D). Increasing the noise the RGC layer allowed the TCC to follow at higher frequencies (σ_OU_ = 30 pA: π/4 point: 12 ± 0.5 Hz; σ_OU_ = 100 pA: π/4 point: 36 ± 0.3 Hz, *P* < 1 × 10^−10^). The fact that increasing noise in the RGC layer allows the TCC layer to respond more rapidly to RGC input is presumably due to the increased synaptic drive the TCC layer receives due to noise driven retinal spikes. This brings TCCs closer to threshold, meaning they need to integrate less stimulus driven input to fire. Thus, the TCC layer fails to produce an accurate representation of sensory input that changes above ~10 Hz.

**Figure 5:**
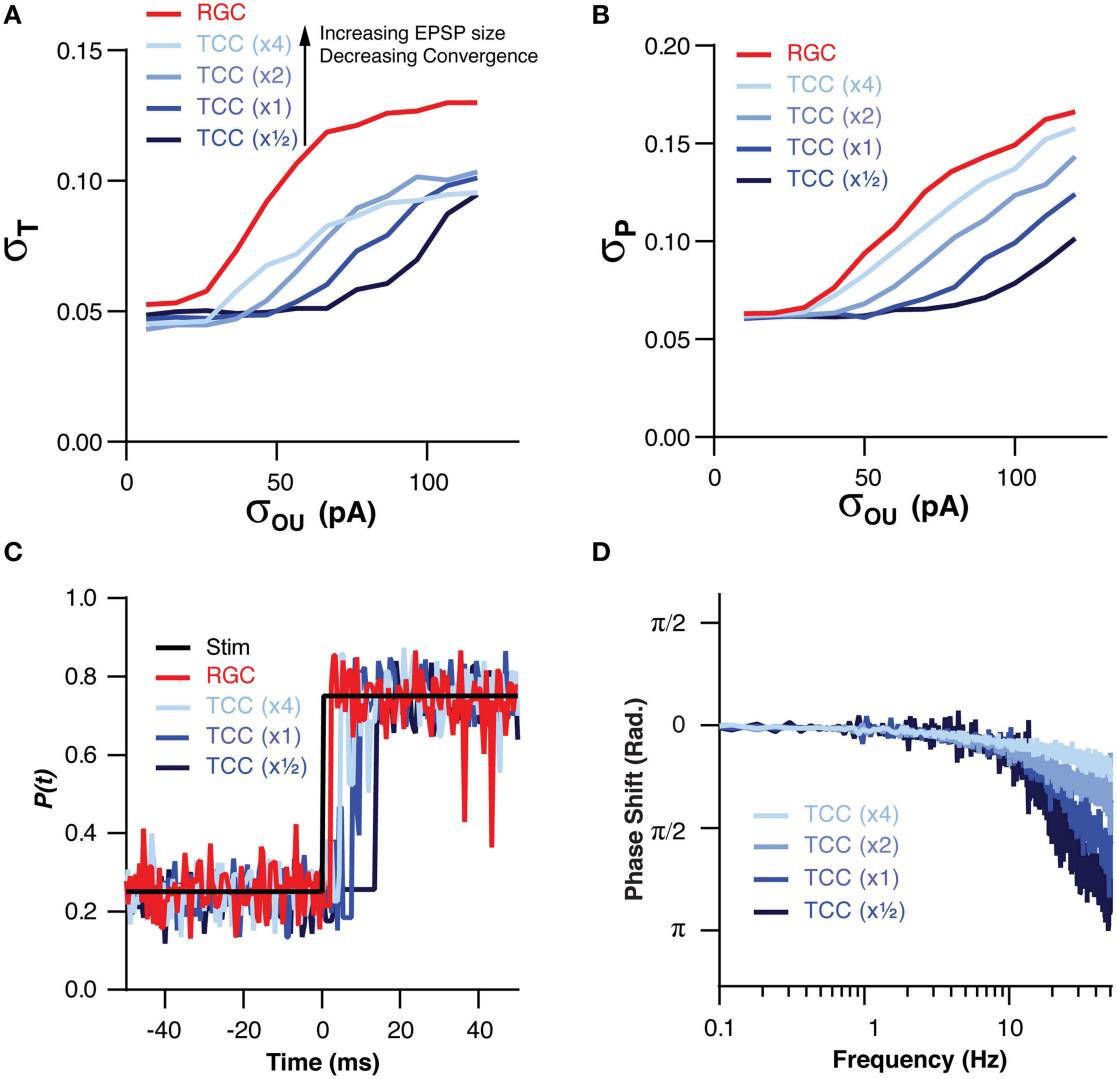
**The low pass effect of the TCC layer is robust to changes in the convergence of RGCs onto TCCs and EPSP size**. **(A)** Across a range of convergence/EPSP sizes, the stability of encoding in the TCC layer was more robust to retinal noise than the RGC layer. Furthermore, increasing the convergence/decreasing the EPSP size produced a greater degree of robustness to noise. **(B)** The accuracy of encoding was higher in the TCC layer than the RGC layer across a range of convergence/EPSP sizes. Again, the higher the convergence/the lower the EPSP size, the greater the robustness to noise was. **(C)** Impulse response of the RGC and TCC layers across a range of convergence/EPSP sizes, showing that response delay increased as the convergence became higher/EPSP size became smaller. **(D)** The phase of the TCC response in relation to the stimuli across a range of convergence/EPSP sizes, clearly showing the slower response of networks with larger convergence and smaller EPSPs.

We have already demonstrated that the filtering effect of the thalamic layer is robust to changes in the numerical ratio of RGC to TCCs, however, we have not investigated how changes in the convergence onto TCCs effects their ability to filter input noise. In order to investigate this we altered the convergence of RGCs onto TCCs in tandem with changing the amplitude of retinal EPSCs. We did this in such a way that an individual TCC might receive, for instance, input from two times as many RGCs but with each one having one half the synaptic weight, thus keeping the total synaptic drive approximately equal. By increasing the convergence of RGCs onto TCCs, the TCC layer became increasingly robust to noise in the RGC cells, both in terms of σ_T_ (*P* = 0.0004) and σ_P_ (*P* < 1 × 10^−7^; Figures 5A,B). However, this came at the cost of increasingly slow frequency response (TC:RGC = 1:1, σ_OU_ = 30 pA, EPSC × 0.5: π/4 point: 6.0 ± 0.5 Hz; EPSC × 4: π/4 point: 60 ± 0.3 Hz; *P* < 1 × 10^−16^; Figures 5C,D). Therefore, across a variety circuit configurations, including ones where single RGC spikes are capable of driving TCC spikes (EPSC × 4), the thalamic layer still acts as a low-pass filter.

## Discussion

Visual acuity is as high, or higher, than the density of RGCs predict (Wässle et al., 1981; Gauthier et al., 2009; Rossi and Roorda, 2010), a fact that may be surprising, given the inherent noise of RGC discharges and the fact that RGCs converge onto TCCs (Croner and Kaplan, 1995). Here we show that TCCs act as a low-pass filter, reducing the consequence of noise driven spikes in the RGCs. However, this behavior comes at a cost: slowing the rate of response.

While this model was explicitly a model of visual system, there is little to suggest the same effect will not be seen in other sensory pathways, as the other sensory pathways share numerous similarities. For instance, input from the trigeminal nuclei to the VPM is also mediated by very large unitary events and that each TCC in the VPM is probably contacted by a small number of trigeminal neurons (Spacek and Lieberman, 1974; Castro-Alamancos, 2002). Furthermore, we have shown that the filtering effect is robust to changes in the underlying connectivity.

Despite the fact that this result was consistent even when model parameters were pushed outside of what has been demonstrated in the native system, one needs to ask whether these results fit with published data. The response of LGN and VPM cells are known to be phase lagged relative to input from RGCs and trigeminal nucleus cells, respectively. However, the reported latency between the RGC and LGN activation (measured as the slope of the phase lag in cycles against frequency) is larger *in vivo* (~15 ms) than reported here (~5 ms), though similar to that reported for trigeminal to VPM (~3–6 ms; Lee et al., 1981; Hartings et al., 2003). Furthermore, there is evidence that thalamic cells do filter sensory noise. Hartings et al. (2003) demonstrated that the noisy (non-modulated) component of the spiking rate of trigeminal nucleus increases almost by a factor of 10 as the rate of whisker stimulation increases, however, the noisy component of the firing rate in VPM neurons is almost unchanged.

We have shown that the TCC layer of our model accurately encodes the information in the RGC layer up to approximately 10 Hz. While this may seem far from optimal, it is worth considering the nature of the information the RGC can provide. Studies in the cat have shown that the retina cannot encode changing information up to arbitrarily high frequencies, and in fact RGC discharges are significant phase shifted relative to visual stimuli above 10 Hz, and have a π/4 point of ~3 Hz (Shapley and Victor, 1978). Thus, the phase shift cause by the TCC may well be minimal in the scope of the visual pathway.

One needs to consider the terminology use in this paper. If we define a channel as the collection of inputs to a network that code for nearly identical stimulus features, then we are describing the thalamic layer as behaving as a low-pass filter in the domain of the rate channel change and not in the domain of rate of input in a given channel. Concretely, a channel could be a collection of trigeminal nucleus neurons that response to a whisker being deflected in a given direction, while another channel would be a collection of neurons who respond when the whisker is deflected in a direction perpendicular to the first direction. Then we are describing the thalamus as limiting the rate at which information on how rapidly the whisker is shifting between moving in those two directions can be transferred, while providing noise immunity and stability of encoding. Indeed, if we consider the domain of the rate of channel change (e.g., the rate of the whisker shifting between moving in two perpendicularly oriented directions), and the domain of the rate of input in a given channel (e.g., mechanosensory current), then these two domains can be thought of almost as inverses, as a stimuli that causes only one channel to be active (low frequency channel change) should cause a consistent drive to a particular set of cells (high frequency input within a given channel). Conversely, a stimuli that is changing rapidly which channels is being driven will drive infrequent input in any given channel. This means that the low-pass behavior of the thalamic layer in the domain of the rate of channel change is a direct consequence of the high-pass behavior of the individual cells in the layer.

The decoding algorithm used here, which was the mean of the centers of the receptive fields of all active cells, and is essentially equivalent to a MLE, was very simple and did not take into account the temporal structure of the neural discharge, or correlation between cells (Usrey et al., 1998; Reinagel and Reid, 2000). This method was chosen because of its simplicity, and the ease of interpreting the results it generates. Moreover, we doubt using a different method would change the fundamental nature of the result: that the thalamic layer will not spike in response to rare input, thereby filtering out noise. This result has been demonstrated in another model (Martinez et al., 2014). However, due to being much closer to the *in vivo* case, this model was far more complex and is applicable to only the visual system.

In conclusion, we have shown that over a wide range of circuit parameters, in terms of changes in the nature of a stimuli, thalamic neurons act as a low pass filter for sensory information. This behavior comes as a consequence of the fact that the cells in the thalamus act as high pass filters in terms of the rate at which they are driven. By only spiking during sustained high frequency input, thalamic cells filter out noise from their inputs allowing higher accuracy and higher stability decode of the stimuli. This however, comes at a cost, in that it produces a delay in transmission of information. We do not propose that this is the only function of the thalamus, but simply that it is a necessary consequence of the fact that TCCs must integrate charge before spiking.

## Author contributions

WC performed simulations and data analysis. ML and AE performed and designed *in vitro* experiments. WC, ML, AE, and VC wrote the manuscript.

## Funding

This work was supported by a Wellcome Trust programme grant (91882, V. Crunelli).

### Conflict of interest statement

The authors declare that the research was conducted in the absence of any commercial or financial relationships that could be construed as a potential conflict of interest.
